# Recanalization and Reperfusion Therapies of Acute Ischemic Stroke: What have We Learned, What are the Major Research Questions, and Where are We Headed?

**DOI:** 10.3389/fneur.2014.00226

**Published:** 2014-11-19

**Authors:** Meritxell Gomis, Antoni Dávalos

**Affiliations:** ^1^Stroke Unit, Neurosciences Department, Hospital Universitari Germans Trias i Pujol, Badalona, Spain

**Keywords:** mechanical thrombectomy, ischemic stroke, stent-retriever, endovascular clinical trials, futile recanalization, reperfusion

## Abstract

Two placebo-controlled trials have shown that early administration of intravenous recombinant tissue plasminogen activator (rt-PA) after ischemic stroke improves outcomes up to 4.5 h after symptoms onset; however, six other trials contradict these results. We also know from analysis of the pooled data that benefits from treatment decrease as time from stroke onset to start of treatment increases. In addition to time, another important factor is patient selection through multimodal imaging, combining data from artery status, and salvageable tissue measures. Nonetheless, at the present time randomized controlled trials (RCTs) cannot demonstrate any beneficial outcomes for neuroimaging mismatch selection after 4.5 h from symptoms onset. By focusing on cases of large arterial occlusion, we know that recanalization is crucial, so endovascular treatment is an approach of interest. The use of intra-arterial thrombolysis was tested in two small RCTs that demonstrated clear benefits in terms of higher recanalization and also in clinical outcomes. But a new paradigm of stroke treatment may have begun with mechanical thrombectomy. In this field, Merci devices have been overtaken by fully deployed closed-cell self-expanding stents (stent-retrievers or “stent-trievers”). However, despite the high rate of recanalization achieved with stent-retrievers compared with other recanalization treatments, the use of these devices cannot clearly demonstrate better outcomes. Thus, futile recanalization occurs when successful recanalization fails to improve functional outcome. Recently, three RCTs, namely synthesis, IMS-III, and MR-rescue, have not been demonstrated any clear benefit for endovascular treatment. Most likely, these trials were not adequately designed to prove the superiority of endovascular treatment because they did not use optimal target populations, vascular status was not evaluated in all patients, relatively high rates of patients did not have enough mismatch, time from baseline neuroimaging to recanalization were too long or the devices used are now obsolete relative to stent-retrievers. Several RCTs currently underway are trying to determine whether bridging therapy is more effective than intravenous treatment and if mechanical thrombectomy is more effective than best medical treatment in patients ineligible for intravenous thrombolysis.

## Introduction

Intravenous recombinant tissue plasminogen activator (rt-PA) is the only approved treatment for acute ischemic stroke within 4.5 h from symptoms onset, as supported by two placebo-controlled trials (1, 2), meta-analyses (3–5), and observational studies (6, 7). However, many patients are still left undertreated due to the fact of factors such as an unknown onset time for the stroke, the narrow time window, a lack of awareness that stroke has occurred or the high number of exclusion criteria for currently approved treatments.

Several strategies have been developed in order to increase the number of treated patients. The EPITHET and DEFUSE studies suggest that multimodal neuroimaging may help to select patients with salvegeable brain tissue up to 6 h after stroke onset (8, 9). EPITHET was a prospective, randomized, double blind, placebo-contolled, and phase II trial that tested alteplase between 3 and 6 h after stroke onset in patients who were imaged with serial echoplanar MRI at baseline and at 3–5 days after therapy. The primary outcome measure was attenuation of infarct growth. Although there was a non-significant trend toward a positive result, in the subgroup of patients with mismatch alteplase was significantly associated with increased reperfusion and improved clinical outcomes (8). The method of measuring this mismatch volume was the standard volumetric technique in which volumes were calculated by simple subtraction. A more precise method based on coregistration of DWI and PWI images showed larger mismatch volumes and significant attenuation of infarct growth by alteplase (9). DEFUSE, an open-label study of intravenous alteplase from 3 to 6 h from stroke onset, showed that the occurrence of early reperfusion led to good clinical outcomes in patients with target mismatch. This mismatch profile was defined as a PWI lesion that was 10 ml or more and 120% or more of the DWI lesion. DEFUSE also identified an MRI pattern called “malignant profile,” characterized by a DWI lesion 100 ml or more and/or a large PWI lesion of 100 ml or more with 8 s or longer of T max delay. The target profile appears to identify patients with an especially robust clinical response rate (67%) after early reperfusion whereas the malignant profile is strongly associated with reperfusion-related brain hemorrhage.

Besides alteplase, new thrombolytic drugs like tenecteplase and desmoteplase may potentially be safer in extended windows up to 6 or 9 h from symptoms onset in patients with salvageable brain tissue. Tenecteplase is a third generation point mutation tissue plasminogen activator that has a longer half-life, greater binding affinity for fibrin and better resistance to inactivation by the endogenous inhibitor PAI-1 compared to alteplase. In a non-randomized study of ischemic stroke patients with CT perfusion mismatch within 3–6 h of symptoms onset, tenecteplase at low dose (0.1 mg/kg) appeared to be superior to alteplase (10). A posterior randomized phase IIb study using a similar design found that moderate (0.25 mg/kg) and low doses (0.1 mg/kg) of tenecteplase yielded significantly better patient outcomes than standard doses of alteplase. Tenecteplase was associated with increased reperfusion, early neurological improvement, and improved 3 month functional outcome with the higher dose without an increase in ICH rate (11).

Desmoteplase is a thrombolytic drug extracted from the saliva of vampire bats. It is more selective for fibrin and has not been to shown to have any deleterious effect on the blood brain barrier compared to alteplase. Two promising phase II studies, DIAS and DEDAS, demonstrated increased reperfusion and strong trends to improved outcome with desmoteplase compared with placebo in acute stroke patients using imaging selection with CT perfusion or multimodal MRI treated within 3–9 h from stroke onset (12, 13). These trial results, however, were not confirmed in DIAS-2 (14). The main reasons for this neutral effect might lie in the lack of standardized imaging assessment of penumbra and the substantial number of patients without main cerebral arterial occlusions. A pooled analysis of DIAS, DEDAS, and DIAS-2 results showed that patients with proximal vessel occlusion or high grade stenosis had greater mismatch and positive response to desmoteplase compared to placebo (15). Current ongoing DIAS-3 and DIAS-4 trials were designed to correct the errors of DIAS-2 (16).

A critical point to obtain a favorable risk/benefit effect of extended intravenous thrombolysis is the existence of an arterial occlusion. A *post hoc* study of pooled data from EPITHET and DEFUSE showed a benefit for intravenous tPA over placebo on infarct growth attenuation only in patients with arterial occlusion (17). Regarding occlusion site, the more distally the occlusion is located, the higher the likelihood of recanalization. By using transcranial Doppler, complete recanalization at 2 h after intravenous rt-PA bolus has been shown in 44, 29, and 10% of cases involving distal middle cerebral artery, proximal middle cerebral artery, and terminal internal carotid artery, respectively (18). However, some adjunctive strategies like sonothrombolysis appear to be safe and obtain higher complete recanalization rates after intravenous thrombolysis. The CLOTBUST-HF study (19) included 20 patients, with 14 of the 20 (70%) occlusions occurring in the middle cerebral artery (12 of these 14 in the proximal middle crebral artery), 3 more (15%) in the terminal carotid artery, and the remaining 3 (15%) in the verebral artery. Rates of complete recanalization at 2 h were 8 out of 20 (40%), all of them MCA occlusions (i.e., 8 or 57% of the 14).

Due to the fact that the effect of tPA is limited in proximal occlusions, other approaches have been tested to open the occluded vessels. Endovascular treatment may be an option to increase recanalization and therefore effective reperfusion rates. A number of trials have tested this hypothesis (Table 1). Endovascular treatment covers two different modalities: intra-arterial administration of thrombolytic drugs, mainly rt-PA and urokinase, and mechanical thrombectomy. Both modalities have the disadvantage over intravenous approaches that they require additional time to be started and are available only in specialized centers. However, the first modality achieves benefits not only because it delivers the drug just into the clot but also from the mechanical effect of the catheter. The PROACT II trial was the first trial to show the benefit of intra-arterial pro-urokinase compared to placebo. Intracranial hemorrhage with neurological deterioration within 24 h occurred in 10% of pro-urokinase patients and 2% of control patients (*p* = 0.06). All symptomatic intracranial hemorrhages occurred in patients with a baseline NIHSS score of 11 or higher (20). Thereafter, the interventional management of stroke (IMS) I and II trials suggested the feasibility and safety of a bridging intra-arterial/intravenous approach compared with intravenous tPA alone. However, the proportion of patients with good clinical outcome (mRS 0–2) at 3 months was only slightly superior in IMS I and II (43 and 46%) compared with patients treated with intravenous tPA in the NINDS trial (39%), although the differences were higher compared with the NINDS placebo subgroup (28%) (21, 22). This treatment modality has since been overtaken by mechanical thrombectomy and in particular by thrombus retrieval.

**Table 1:** **Baseline stroke severity and outcome variables in the main reperfusion trials carried out before the development of stent-retrievers**.

	*n*	Baseline NIHSS	Successful recanalization (%) (TIMI 2–3)	mRS 0–2 at 90 days (%)	90-day mortality (%)	sICH (%)
**Intravenous thrombolysis**
Pooling analysis of phase IV trials within 6 h (tPA groups) (4)	1391	11	NA	49	13	5–9^b^
Pooling analysis of phase IV trials within 6 h including IST-3 (tPA groups) (5)	3548	–	NA	46	19	7^b^
**Endovascular treatment**
PROACT II (20)	121	17	66	40	25	10
IMS (21)	62	18	56	43	16	6
IMS-II (23)	55	19	58	46	16	10
MERCI (24)	141	20	48^a^/60	28	44	8
Multi MERCI (25)	164	19	55^a^/68	36	34	10
Penumbra (26)	125	18	82	25	33	11
**Control groups**
Pooling analysis of phase IV trials within 6 h (placebo groups)	1384	11	NA	44	15	1.1^a^
PROACT-II (control group)	59	17	18	25	27	2

*^a^Device alone*.

*^b^Parenchimal hematoma type II. sICH, symptomatic Intra-cerebral Hemorrhage; tPA, tissue plasminogen activator; mRS, modified Rankin Scale; NIHSS, National Institutes of Health Stroke Scale*.

Mechanical approaches compared to intra-arterial pharmacological therapy are associated with greater technical difficulty, the potential development of vasospasm, vessel dissection, perforation or rupture, and distal embolization into previous unaffected territories due to fragmented thrombus. The MERCI^®^ device achieved complete recanalization in 48% of acute ischemic stroke patients with large arterial occlusions treated within the first 8 h from stroke onset (24). The MERCI trial (Table 1) included a large number of patients who were not eligible for intravenous tPA. Their results were comparable with the results of PROACT-II (20) and IMS-II (23). Conversely, mortality was higher in the MERCI trial in accordance with the differences between populations. Arterial recanalization was independently associated with good outcome (mRS ≤2 at 3 months), while the absence of recanalization was associated with mortality. Although the recanalization rate with mechanical thrombectomy was similar to that for intra-arterial thrombolysis, only 25% of MERCI patients achieved a good functional outcome. In the Multi MERCI trial (25) (Table 1), investigators used a new generation of MERCI^®^ devices (L5 Retriever) in patients with large-vessel stroke within 8 h of symptoms onset. Regarding safety, no differences were seen in the rates of intracranial hemorrhage or clinically significant procedural complications between those patients treated with intravenous tPA and those who were not. Another single-center study obtained similar findings (27). A new thrombectomy system was used in the Penumbra Trial, designed to evaluate the safety and effectiveness of the Penumbra thrombo-aspiration device (26) (Table 1) in patients with acute ischemic stroke within the 8 h from symptoms onset with NIHSS ≥8 and angiograhic occlusion. Procedural events occurred in 12.8% of cases, with 2.4% considered serious. In this study, 25% of patients achieved a modified Rankin Scale score of ≤2. Taken together, these findings suggest that the Penumbra System obtains higher revascularization rates than those reported for the MERCI^®^ device. The safety profile of the Penumbra System is also favorable but despite this, considering the high revascularization rate, the clinical functional outcome was lower than expected. The absence of imaging-guided patient selection and historical control design in these studies may render elusive a definitive conclusion on long-term outcome. Therefore, the effect of revascularization on clinical functional outcome should only be evaluated by a controlled trial in well-selected patients.

Recently, a new paradigm of more promising stroke treatment has begun with the use of fully deployed closed-cell-expanding stents (stent-retrievers or “stent-trievers”), which achieve recanalization rates of up to 90%. However, improvement of revascularization has not been paralleled by favorable responses in clinical outcome. DEFUSE 2 (28) results for which have only been published recently, was a prospective cohort study that enrolled patients to have endovascular treatment within 12 h from symptoms onset to establish whether the patient had an MRI baseline profile (target mismatch) predictive of salvageable tissue. A total of 46 out of 78 (59%) patients with target mismatch and 12 out of 21 (57%) patients without target mismatch had reperfusion after endovascular treatment. The adjusted odds ratio (OR) for favorable clinical response associated with reperfusion was 8.8 [95% confidence interval (CI) 2.7–29.0] in the target mismatch group and 0.2 (0.0–1.6) in the no target mismatch group (*p* = 0.003 for difference between ORs). Reperfusion was associated with increased good functional outcome at 90 days (OR 4.0; 95% CI 1.3–12.2) in the target mismatch group, but not in the no target mismatch group (1.9; 0.2–18.7). Thus, this trial showed that target mismatch patients who had early reperfusion after endovascular treatment had more favorable clinical outcomes. No association between reperfusion and favorable outcomes was present in patients without target mismatch.

In the overview that follows, we will analyze the limitations of previous research and discuss future trial designs and as well as the prospects for endovascular treatment for acute ischemic stroke patients.

## Endovascular Treatment for Acute Ischemic Stroke

### What have we learned?

Recanalization continues to be one of the most powerful predictors of successful outcome and is used as a surrogate of efficacy in acute stroke RCTs. In a formal meta-analysis (29) involving a total of 2066 patients, reported recanalization rates categorized according to the received intervention were spontaneous in 24.1% of cases, 46.2% after intravenous thrombolysis, 63.2% after intra-arterial thrombolysis, 67.5% after combined intravenous-intra-arterial, and 83.6% after mechanical thrombectomy. Clinical outcome categorized by as either success or failure in achieving recanalization was available from 998 patients. Good functional outcomes at 3 months were more frequent in recanalized vs. non-recanalized patients with OR of 4.43 (95% CI 3.32–5.91). Three-month mortality was reduced in recanalized patients (OR 0.24; 95% CI 0.16–0.35). Rates of symptomatic hemorrhagic transformation did not differ between the two groups (OR 1.11; 95% CI 0.71–1.74). These findings thus suggest that recanalization is an appropriate biomarker of therapeutic activity in early phase trials of revascularization therapies in acute ischemic stroke.

Although arterial recanalization and subsequent reperfusion should allow the restoration of brain function when it is done early after ischemic stroke, this often fails. This futile recanalization has been related to multiple downstream embolization, the non-reflow phenomenon caused by blockage of microcirculation or fast recruitment of ischemic tissue into infarction before recanalization. Furthermore, reperfusion can be deleterious, through brain–blood barrier disruption resulting in massive brain edema or hemorrhagic transformation (30). Specifically, Hussein et al. (31) in a multicentre study observed futile recanalization in 49% of patients who received endovascular treatment for acute ischemic stroke. Age >70 years (OR 4.4; 95% CI 1.9–10.5) and initial NIHSS from 10 to 19 (OR 3.8; 95% CI 1.7–8.4; *p* < 0.001) emerged as independent predictors of futile recanalization.

### *Stent-Retrievers* 

As noted above, removable cerebral stents and clot retriever devices referred to as stent-retrievers are a promising strategy to reinforce mechanical thrombectomy. These devices achieve high rates of recanalization and avoid the hemorrhagic complications associated with the use of antithrombotic drugs needed when an angioplasty and permanent stenting is used to achieve arterial recanalization (32).

#### Retrospective non-controlled non-randomized studies

The first reported cases dealt with permanent placement of open-cell self-expanding stents to recanalize embolic intracranial artery occlusions by compressing the occluding thrombus represented a big step forward in mechanical thrombectomy (33, 34). Multiple small case series reporting the results of the use of stent-trievers have been published. Most of them showed high rates of recanalization and outcome rates comparable with those reported in intravenous thrombolysis trials. In general, patients treated with these devices had higher NIHSS and were treated later than patients treated with intravenous thrombolysis (35–57) (Table 2).

**Table 2:** **Comparision of baseline stroke severity and outcome variables in studies using stent-retrievers**.

Author/year	*n*	Baseline NIHSS median or mean ± SD	Successful recanalization (%) (TIMI 2–3)/(TICI 2b-3)	mRS 0–2 at 90 days (%)	90-day mortality (%)	sICH (%)
**Retrospective non-controlled non-randomized studies**
Castaño et al. (54)^b^	1	23	100	100 mRS 3	0	0
Suh et al. (35)^b^	1	15	100	–	–	0
Castaño et al. (36)	20	19	90	45	20	10
Roth et al. (44)	22	19.4 ± 5.7	90.9	50	18.1	9
Venker et al. (46)	10	15.6 ± 5.3	100	–	–	0
Cohen et al. (37)	6	>17	100	100	0	0
Rohde et al. (43)	10	19	100	60 (30 days)	30	20
Stampfls et al. (45)	18	21 ± 6.7	88.8	33,3	27.7	16.6
Wehrschvetz et al. (47)	11	16 ± 4.7	18	30	9	0
Miteff et al. (48)	26	–	96	42	19	10
Costalat et al. (53)	50	15	84	54	12	2
Park et al. (52)	8	–	100	–	–	0
Pérez et al. (42)^b^	1	10	100	100 (30 days)	–	0
Kim et al. (34)	14	10	78.6	57.1	–	7.1
Cohen et al. (38)	17	>12	100	88.2 at month	–	11.8
Machi et al. (39)	56	16	89.2	46.4 discharge	7.1	1.7
Möhlenbruch (41)	25	–	88	–	–	12
Castro Alfonso (48)	21	17 ± 6.36	90.1	61.9	9.5	14.2
Menon et al. (51)	40	–	85.7	57.1	14.3	–
San Roman et al. (50)^a^	60	18	86.7	45	28	12
Mpotsaris et al. (56)	26	16	88	38	7.6	–
Dávalos et al. (55)	141	18	85	55	20	4
Mendonça et al. (40)	13	19	77	30	30	0
**Prospective studies with independent evaluation**
STAR (57)	202	17	79.2	57.9	6.9	1.5
TREVO (58)	60		78.3	55	20	5
**Prospective randomized crontrolled studies**
SWIFT (Solitaire) (59)	58	18	61	58	17	2
	55	18	24	33	38	11
TREVO-2 (Trevo) (60)	88	19	86	40	33	7
	90	18	60	22	24	9

*sICH, symptomatic intra-cerebral hemorrhage; mRS, modified Rankin Scale; NIHSS, National Institutes of Health Stroke Scale*.

*^a^Prospective study*.

*^b^Single case reports*.

#### Prospective controlled studies

The solitaire flow restoration thrombectomy for acute revascularization (STAR) was a prospective, multicentre, single-arm study of mechanical thrombectomy using the Solitaire device (58) that included 202 patients with a median basal NIHSS score of 17. Successful recanalization was achieved in 79.2% of patients and favorable neurological outcome in 57.9%. Procedure-related complications occurred in 7.4%, intracranial hemorrhagic transformation of some sort occurred in 18.8%, with 1.5% being symptomatic, and mortality at 3 months occurred in 6.9%. A similar study was conducted with the TREVO stent-retriever. The Trevo^®^ study (59) was a prospective, multicentre, and single-arm study in acute stroke patients in which a total of 60 patients were enrolled. A TICI 2b-3 was achieved in 78.3%. At 90 days, 55% of the patients had a favorable neurological outcome (mRS 0–2) and 20% had died. Patients with successful recanalization (TICI 2a, 2b, and 3) had a 60% rate of good neurological outcome at day 90 (mRS 0–2), whereas no patient without recanalization had a mRS 90 < 3. The overall rate of symptomatic intra-cerebral hemorrhage according to the SITS-MOST criteria was 5% (3/60) (Table 2).

#### Prospective randomized controlled studies

Solitaire^TM^ AB/FR and Trevo^®^ were compared with the standard predicate mechanical thrombectomy device, the Merci Retrieval System, in two controlled randomized trials (SWIFT and TREVO-2) to test the potential superiority of stent-retrievers. SWIFT (60) was a randomized, parallel-group, non-inferiority trial that randomly allocated 58 patients to the Solitaire group and 55 patients to the Merci group. The primary endpoint was partial or complete recanalization (thrombolysis in myocardial ischemia, TIMI 2 or 3) without symptomatic intra-cerebral hemorrhage, assessed by an independent CoreLab, which was masked to study assignment. Primary analysis was done by intention to treat. The primary efficacy outcome was achieved more often in the Solitaire group than in the Merci group (61 vs. 24%; difference 37% [95% CI 19–53], OR 4.87 [95% CI 2.14–11.10]; *p* non-inferiority <0.0001, *p* superiority = 0.0001). More patients had 3 months good neurological outcome in the Solitaire group than in the Merci group (58 vs. 33%; difference 25% [95% CI 6–43], OR 2.78 [95% CI 1.25–6.22]; *p* non-inferiority = 0.0001, *p* superiority = 0.002). Ninety-day mortality was lower in the Solitaire group (17 vs. 38%; difference −21% [95% CI −39 to −3], OR 0.34 [95% CI 0.14–0.81]; *p* non-inferiority = 0.0001, *p* superiority = 0.02). These findings confirm that Solitaire achieves better angiographic results and clinical outcomes than does the Merci Retrieval System. TREVO 2 trial (61) was an open-label randomized controlled trial that included patients aged from 18 to 85 years with confirmed large-vessel occlusion stroke and NIHSS score from 8 to 29 within 8 h from symptoms onset. Randomization was stratified by age (≤68 vs. 69–85) and NIHSS scores (≤18 vs. 19–29). The primary efficacy endpoint was thrombolysis in cerebral infarction (TICI) scores ≥2 assesed by an ummasked CoreLab. The primary safety endpoint was a composite of procedure-related adverse events. Analyses were done by intention to treat. The TREVO 2 trial randomly allocated 88 patients to the Trevo group and 90 patients to the Merci group. Seventy-six patients (86%) in the Trevo group and 54 (60%) in the Merci group met the primary endpoint (OR 4.22; 95% CI 1.92–9.69; *p* superiority <0.0001). Incidence of the primary safety endpoint did not differ between groups [13 (15%) patients in Trevo group vs. 21 (23%) in the Merci group; *p* = 0.1826]. However, vessel perforations were almost 10 times more common with Merci devices (10%) than with the Trevo retriever (1%; *p* = 0.0182). Notably, these perforations did not seem to have high clinical relevance, since the rates of symptomatic intracranial hemorrhages and periprocedural mortality were similar in the two groups (Table 2).

A single-center, prospective study on 33 patients showed no significant differences between the Trevo and Solitaire stent-retrievers (62).

The results of these trials were encouraging and support the use of stent-retrievers in prospective trials of endovascular treatment against medical treatment alone.

### *Controlled trials of endovascular treatment* 

Three randomized clinical trials (IMS-III, SYNTHESIS, and MR-RESCUE) (63–65) designed to prove the superiority of endovascular treatment compared to intravenous rt-PA in acute ischemic stroke failed to demonstrate any benefits. We briefly describe the main characteristics and results of these trials, and point out the weaknesses that probably determined the negative results.

The IMS-III (63) was a randomized, parallel-arm trial comparing intravenous tPA followed by endovascular treatment with intravenous tPA alone in patients with acute ischemic stroke within 3 h from symptoms onset. The angiographic procedure had to begin within 5 h and be completed within 7 h after stroke onset. The trial intended to enroll 900 subjects to ensure adequate statistical power to detect an absolute 10% difference in the percentage of patients with good outcome (mRS 0–2) at 3 months. After 656 patients were randomized (434 participants to endovascular therapy and 222 to intravenous tPA alone), the study was prematurely stopped based on the pre-specified criterion for futility. The proportion of patients with a modified Rankin score of 2 or less at 90 days did not differ significantly according to treatment (40.8% with endovascular therapy and 38.7% with intravenous tPA alone; absolute difference, 1.5 percentage points; 95% CI −6.1 to 9.1, after adjustment for the baseline NIHSS score. There were no significant differences between the two treatment arms in the pre-defined subgroups of patients with a NIHSS score ≥20 (6.8 percentage points; 95% CI, −4.4 to 18.1) and ≤19 (−1.1 percentage point; 95% CI −10.8 to 8.8). Both groups showed similar mortality rates at 90 days (19.1 and 21.6%, respectively; *p* = 0.52) and symptomatic intra-cerebral hemorrhage within 30 h after initiation of tPA (6.2 and 5.9%, respectively; *p* = 0.83).

In the SYNTHESIS trial (64), 362 patients with acute ischemic stroke of less than 4.5 h after symptoms onset were 1:1 randomly allocated to receive intravenous tPA or endovascular therapy (intra-arterial thrombolysis with tPA, mechanical clot disruption or retrieval, or a combinaton of these approaches) within <6 h from onset. Primary outcome was survival free of disability at 3 months, defined as modified Rankin score 0–1. The median time from stroke onset to start of treatment was 3.75 h for endovascular therapy and 2.75 h for intravenous tPA (*p* < 0.001). Good primary outcome was found in 30.4% of patients in the endovascular treatment group and in 34.8% in the intravenous group (OR adjusted for age, sex, stroke severity, and atrial fibrillation status at baseline 0.71; 95% CI 0.44–1.14; *p* = 0.16). Symptomatic intracranial hemorrhage within 7 days occurred in 6% of patients in each group; there were no differences between groups in the rate of other serious adverse events or mortality. Subgroup analysis suggested that the lack of superiority of endovascular therapy was not explained by the time delay to endovascular treatment, stroke subtype, or center. Importantly, the demonstration of vessel occlusion was not a precondition for inclusion in this trial and the use of mechanical thrombectomy devices was limited to Solitaire in 18 patients, Penumbra in 9, Trevo in 5, and Merci in 5.

The MR RESCUE trial (65) was designed to study whether brain imaging could identify patients who were most likely to benefit from therapies for acute ischemic stroke and whether endovascular thrombectomy improved clinical outcome. The trial included patients within 8 h after the onset of large-vessel, anterior circulation strokes that were randomly assigned to undergo mechanical embolectomy (Merci retriever or Penumbra System) or medical treatment. Randomization was stratified according to whether the patient had a favorable penumbral pattern (substantial salvageable tissue and small infarct core) or a non-penumbral pattern (large core or small or absent penumbra). Among 118 eligible patients, the mean time to enrollment was 5.5 h, and 58% had a favorable penumbral pattern. Revascularization in the embolectomy group was achieved in 67% of patients, 90-day mortality was 21% and the rate of symptomatic intracranial hemorrhage was 4%; no rate differed across groups. Among all patients, the mean score on the modified Rankin score was equal in the embolectomy and standard of care groups (3.9 vs. 3.9, *p* = 0.99). Embolectomy was not superior to standard of care in patients with either a favorable penumbral pattern (mean score 3.9 vs. 3.4; *p* = 0.23) or a non-penumbral pattern (mean score 4.0 vs 4.4; *p* = 0.32). There was no interaction between the pretreatment imaging pattern and treatment assignment on the favorable primary outcome effect (*p* = 0.14). There was a relatively low rate of substantial revascularization in the embolectomy group, which could be related to the use of first-generation embolectomy devices. Long delays up to 5.3 ± 1.6 h from imaging to embolectomy and the heterogeneity of imaging approaches based on the use of both MRI and CT were additional factors that might have diluted treatment effect. A favorable penumbral pattern beyond 3 h may be a signature of more vigorous collateral vessels and therefore of greater tolerance to occlusion, increased likelihood of spontaneous recanalization and good outcome (66). The MR Rescue trial did not show a differential benefit among patients who underwent embolectomy between those with a favorable penumbral pattern as compared with those with a non-penumbral patttern. These findings differ from those in the DEFUSE 2 trial where patients had a shorter time until treatment and smaller predicted infarct cores.

IMS-III and SYNTHESIS did not target the best patient populations to achieve positive results. These trials failed to prove the superiority of endovascular treatment because vascular status was not systematically evaluated, salvageable brain tissue was presumably small or not present in many patients, time from baseline neuroimaging to recanalization was too long and the devices used have been rendered obsolete by stent-retrievers. Despite these limitations, these trials have demonstrated that endovascular treatment is safe and provide data that could be relevant in the design of future trials aiming to prove the applicability of this treatment in certain patients (67–69).

Concerning reperfusion scales, the Thrombolysis in Myocardial Infarction Scale (TIMI), originally constructed to measure myocardial reperfusion, has been widely adopted for use in cerebral circulation (Table 3) (70). The TICI Scale was originally proposed in a position statement that attempted to standardize clinical trial design and reporting for intra-arterial therapy. The TICI scale specifically addresses the extent of tissue reperfusion, as represented by the capillary blush on digital subtraction angiography (71). The original TICI system defined TICI 2b as restoration of more than two thirds of the target downstream territory. This is in contrast to the subsequent modified version mTICI (modified treatment in cerebral ischemia introduced by the IMS investigators, which uses a threshold of more than half of the target downstream territory (22, 23) (Table 4). In a comparative study of TIMI and mTICI, the c-statistic for predicting 90-day good outcome (mRS 0–2) was significantly higher for mTICI vs. TIMI (0.74 vs. 0.68; *p* < 0.0001) (72). Currently, the target angiographic endpoint for assigning technical success should be mTICI 2b or higher (73).

**Table 3:** **Thrombolysis in Myocardial Ischemia Scale**.

TIMI grades	Definitions
Grade 0	Absence of any antegrade flow beyond the target occlusion (no perfusion)
Grade 1	Any faint antegrade flow beyond the target occlusion, with incomplete filling of the distal branches (penetration without perfusion)
Grade 2	Delayed or sluggish antegrade flow with complete filling of the distal M2 branches flow (partial perfusion)
Grade 3	Normal flow that fills all distal branches, including M3 and M4 (complete perfusion)

*TIMI indicates thrombolysis in myocardial ischemia*.

**Table 4:** **Modified treatment in Cerebral Ischemia Scale**.

mTICI grades	Definitions
Grade 0	No perfusion
Grade 1	Antegrade reperfusion past the initial occlusion, but limited distal branch filling with little or slow distal reperfusion
Grade 2a	Antegrade reperfusion of less than half of the occluded target artery previously ischemic territory (e.g., in one major division of the MCA and its territory)
Grade 2b	Antegrade reperfusion of more than half of the previously occluded target artery ischemic territory (e.g., in two major divisions of the MCA and their territories)
Grade 3	Complete antegrade reperfusion of the previously occluded target artery ischemic territory, with absence of visualized occlusion in all distal branches

*MCA indicates middle cerebral artery; and mTICI is modified treatment in cerebral ischemia scale*.

In summary, we have learned from these trials that endovascular treatment after tPA is as safe as endovascular treatment alone and that stent-retrievers achieve faster arterial recanalization in acute ischemic stroke. We also have learned that while endovascular therapy may only be useful for selected patients, who we do not yet know how to select optimally, it has not proven useful for stroke patients in general. Thus, it is essential to use the lessons of these trials results in order to design new trials.

### How can we advance?

Randomized controlled trials (RCTs) need to answer a set of open questions. It is very important that they all move together in the same direction and randomize all the eligible patients in order to avoid selection bias. We may make progress through the challenges that a knowledge of previous studies affords us.

#### Concerning the endovascular treatment arm of the trials

Recent neutral RCTs of endovascular therapy have shown that the technological improvement of devices moves faster than patient recruitment. Consequently, ongoing and future trials should be inclusive and allow the use of any approved device that interventionalists believe will yield the best results. The main problem with this approach is that different devices or techniques may have different complications and efficacy rates, so merging devices may potentially affect the interpretation of the endovascular arm of the trials.

“Time is brain,” so any time delay in treatment administration must be minimized. As in any trial, endovascular stroke trials require mandatory steps that are time-consuming such as obtaining informed consent, checking inclusion, and exclusion criteria and randomization. Therefore, it is important to reduce time delays affecting the endovascular treatment arm through randomization in the angiosuite with team activated, and with a precisely definined standard metric for door-to-groin-puncture time (74). Moreover, these trials might benefit from close cooperation between the different participating centers in order to shorten the delay caused by the transfer of patients.

Centers with best and fastest endovascular stroke treatment facilities should be preferentially considered for such trials, and it is important to carry out extensive preparation and continued education and monitoring of all participating centers to ensure their ability to randomize patients appropriately and achieve as short a time as possible from imaging to reperfusion. Problems generated by this approach are slow recruitment and lack of generalizability of the trial results. Regarding standarization of the procedure, in general, precise metrics need to be defined and monitored. It is also important to have a set of pre-defined actions that should be taken when the center being monitored does not fullfill the required metrics.

There is consensus that, if possible, general anesthesia should be avoided due to the fact that it is associated with worse functional outcome and time delays (75).

#### Concerning imaging-based patient selection

There is no doubt that CT or MR imaging including the study of the ischemic core and vessel occlusion is highly recommended for endovascular patient selection. Collateral status has emerged as critical for brain tissue survival until the clot is lysed and sufficient anterograde perfusion is achieved, so an emerging challenge is obtaining additional information about collateral status in endovascular trials (76–79). The Safety and Efficacy of Neuroflo Technology in Ischemic Stroke (SENTIS) trial was the first randomized controlled trial to test the effect of a device to potentially increase collateral blood flow to the brain (80). Although the trial failed to meet the pre-specified primary efficacy endpoint, the safety of the treatment was confirmed. Also, it showed that patients with favorable vascular profiles in multimodal imaging were most likely to benefit from this approach, mainly in the older cohort of patients above 70 years of age (81). Analysis of the IMS III trial results showed that robust angiographic collateral grade was a significant predictor of good clinical outcome at 90 days. Similar findings were seen in the SWIFT and TREVO2 trials. Thus, it appears that collateral evaluation may be used to enhance the approach to treat stroke and to refine future trial designs.

#### Concerning trial design

Probably the most important problem for the internal validity of a trial is when a substantial number of eligible patients are presumibily treated outside of the trial. A powerful potential challenge would be to use government-mandated and audited population-based databases of reperfusion therapies (82).

The main outcome based on the degree of functional disability is usually measured by the modified Rankin Score. This score is a monotonic scale, except for the similarity between the two most severe levels, 5 and 6 (83). As a consequence, a “shift” analysis is more likely to find relevant differences in the modified Rankin Score distribution between the two groups in a study than a pre-specified threshold.

#### Currently, there are two major research questions

##### Is bridging therapy more effective than intravenous treatment in patients with large-vessel occlusions in the anterior circulation?

Bridging therapy is safe but the question about whether it is more effective than intravenous treatment remains open. Trials should compare intravenous thrombolytic treatment as the control group vs. intravenous thrombolysis plus mechanical thrombectomy as the experimental group. A trial comparing intravenous tPA vs. mechanical thrombectomy in patients eligible for intravenous thrombolysis could be a reasonable design once combined therapy had been demonstrated to be superior to intravenous tPA.

Bridging therapy might be better than isolated intravenous tPA in patients with proximal vessel occlusions. Trials comparing the two treatment arms should include a baseline imaging vessel study. A rather small proportion of patients in IMS III trial had proximal occlusions. In this subgroup of patients combined treatment was superior to intravenous tPA alone (84).

##### Is mechanical thrombectomy more effective than best medical treatment in patients ineligible for intravenous thrombolysis?

Trials comparing endovascular treatment vs. best medical treatment without administration of intravenous tPA may be more difficult to carry out since many physicians may consider this design unethical despite the fact that there are no RCTs demonstrating the superiority of endovascular treatment in these situations.

There are several reasons for considering a patient ineligible for intravenous thrombolysis, which can be divided into two different profiles: those that derive from limits in the time window and those that are associated with a higher risk of bleeding such as abnormal hemostasis, anticoagulant treatment with an INR higher than 1.7 or recent (last two months) major surgery. These two groups may have different safety profiles.

Patients arriving for treatment beyond 4.5 h are ineligible for intravenous rt-PA. Endovascular treatment, mainly mechanical thrombectomy, could be a good alternative treatment but currently there are no results from RCTs answering this open question. In these longer time windows, good clinical outcome is associated with the presence of salvageable brain tissue, which mainly depends on collateral flow status, which in turn may be estimated by means of different imaging modalities, e.g., ASPECTS score, CT angiography source imaging, CT perfusion, or multimodal MRI. A retrospective analysis of one multicentre prospective cohort study (DEFUSE 2) suggested that MRI could diagnose patients who did or did not benefit from the treatment before interventional treatment up to 12 h from symptom onset. These data need to be confirmed in a randomized study but suggest that the time window for treatment can be extended in some patients. In the Penumbra pivotal stroke trial (27), recanalization benefited patients with a favorable image on the baseline CT scan as indicated by an ASPECTS score >7. In fact, some patients are able to maintain a penumbra for as long as 48 h (79, 85) and thus may still benefit from mechanical thrombectomy. Natarajan et al. (86) published a study showing the benefit with safety of endovascular therapy in patients after 8 h from symptoms onset and wake-up strokes. For these reasons, the use of imaging selection criteria is essential in these studies.

Focusing on the other group of patients, out of the three causes, patients with illness producing abnormal hemostasis are anecdotal. More common are patients undergoing oral anticoagulant treatment, mostly with anti-vitamin K drugs. In these cases, although only short series are reported in the literature, the results are quite good (87). Patients with recent surgery can not receive endovenous tPA due to the risk of bleeding. These patients can suffer a stroke mediated by a procoagulant state and sometimes favored by the withdrawal of antiplatelets or anticoagulants. Data from several case series have been published showing that the endovascular approach is safe and effective (88).

### Where are we headed?

Several prospective and randomized clinical trials of mechanical thrombectomy are now enrolling patients to overcome the limitations of previous trials.

The main ongoing randomized clinical trials for mechanical thrombectomy are THRACE, PISTE, MRCLEAN, and REVASCAT in Europe, SWIFT-PRIME in the US, and THERAPY and ESCAPE in Canada (Table 5).

**Table 5:** **Characteristics of main ongoing randomized controlled clinical trials of endovascular treatment**.

	THRACE French	MRCLEAN Netherland	PISTE UK	REVASCAT Spanish	SWIFT PRIME US	THERAPY US	ESCAPE Canada
PI	S. Bracard, X. Ducroq	C. Majoie	K. Muir, P. White	A. Dávalos	J. Saver, C. Diener, E. Levy, V. Pereira	J. Mocco, P. Kathri	MD Hill
Random	MT (solitaire,Merci, catch, penumbra) +IV tPA vs. IV tPA	Endovasc. treatment vs. IV tPA	All devices for mechanical thrombectomy vs. IV tPA	Solitaire vs. best medical treatment	IV tPA+ solitaire FR vs. IV tPA	IV tPA + Penumbra vs. IV tPA	Endovasc. thrombectomy vs. best medical treatment
Hypothesis	Superiority (15%)	Non-inferiority	Superiority (15%)	Superiority (15%)	Superiority (10%)	Superiority (12%)	Superiority (20%)
Sample size	480	500	400	690	Up to 941 (expected 600)	692	242
IV tPA	4 h	4.5 h	4.5 h	4.5 h	4.5 h	4.5	4.5 h
EVT	<5 h	<6 h	<6 h	<8 h	<6 h	<4.5	<12 h
Primary efficacy endpoint	mRS at 90 days	mRS at 90 days	mRS at 90 days	Rate of mRS 0–2 at 90 days	Reduced stroke-related disability (mRS 90 days)	Rate of mRS 0–2 at 90 days	NIHSS 0–2 or mRS 0–2 at 90 days

*PI, principal investigator; mRS, modified Rankin Scale; NIHSS, National Institutes of Health Stroke Scale; MT, mechanical thrombectomy; EVT, endovascular treatment; IV tPA, intravenous tPA*.

Trial and cost-effectiveness evaluation of intra-arterial thrombectomy in acute ischemic stroke (THRACE) (ClinicalTrials.gov Identifier: NCT01062698) is a multicentre randomized controlled trial with the primary objective of determining whether a combined approach of intravenous thrombolysis (IV) plus mechanical thrombectomy (Merci, Penumbra, Catch, and Solitaire) is superior to IV thrombolysis alone within the 3 h of onset of symptoms in patients with occlusion of proximal cerebral arteries and a NIHSS ≥10. The secondary aim is to determine the cost-effectiveness of this procedure compared to the standard treatment (IV thrombolysis). Projected sample size is 480 patients. At the present time, the recruitment criteria for this study is unknown because the information has not been verified.

Pragmatic Ischemic Stroke Thrombectomy Evaluation [(PISTE); A randomized controlled clinical trial of adjunctive mechanical thrombectomy compared with intravenous thrombolysis in patients with acute ischemic stroke due to an occluded major intracranial vessel] (ClinicalTrials.gov Identifier: NCT01745692) is a randomized controlled trial testing whether mechanical thrombectomy improves functional outcome in patients with large artery occlusion on top of IV thrombolysis. The projected sample size is 800 subjects. This study is not yet open for participant recruitment.

Multicenter Randomized Clinical trial of Endovascular treatment for Acute ischemic stroke in the Netherlands (MR CLEAN) (ISRCTN10888758) is a pragmatic phase III multicentre randomized clinical trial with blinded outcome assessment. The primary objective of this study is to estimate the effect of endovascular treatment on overall functional outcome after acute ischemic stroke of less than 6-h duration in patients with a symptomatic intracranial anterior circulation occlusion. Intervention arm consist of endovascular treatment by means of the local application of rt-PA or urokinase by guided micro-catheter, and/or mechanical thrombectomy, by means of a retraction device, aspiration device, or retrievable stent. The intervention arm could be for patients who have been treated in successfully with IV thrombolysis, patients who can be treated within 6 h but do not meet the time window requirements for IV thrombolysis, and patients with contraindications for IV or intra-arterial thrombolytic treatment (thrombectomy only). The control arm consists of regular treatment according to current national clinical guidelines, including intravenous t-PA within the first 4.5 h after symptom onset. The projected sample size is 500 subjects. Recruitment has recently finished.

Endovascular Revascularization With Solitaire Device vs. Best Medical Therapy [(REVASCAT) in anterior circulation stroke within 8 h] (ClinicalTrials.gov Identifier: NCT01692379) is a prospective, multicentre randomized trial seeking to establish whether subjects meeting the main inclusion criteria of age 18–85, baseline NIHSS ≥6, evidence of TICA or proximal (M1) MCA, ASPECTS score of ≥7 on NCCT or ≥6 on diffusion-weighted MRI, ineligible for or with persistent occlusion after IV alteplase and treated within 8 h from symptom onset have higher rates of favorable outcome when treated with the Solitaire embolectomy device compared to standard medical therapy alone. The primary endpoint, based on intention-to-treat criteria, is the distribution of mRS scores at 90 days. Maximum sample size is 690 patients with three previous specified interim looks. Randomization is performed under a minimization process using age, baseline NIHSS, therapeutic window, occlusion location, and investigational center. Secondary endpoints are infarct volume evaluated on CT at 24 h, dramatic early favorable response, defined as NIHSS of 0–2, or NIHSS improvement ≥8 points at 24 h and successful recanalization in the Solitaire arm according to the TICI classification defined as TICI 2b or 3. Safety variables are mortality at 90 days, symptomatic intracranial hemorrhage rates at 24 h, and procedure-related complications.

Endovascular treatment for small core and proximal occlusion ischemic stroke (ESCAPE) (ClinicalTrials.gov Identifier: NCT01778335) is a phase III, randomized, open-label with blinded outcome evaluation, controlled, parallel-group trial. The primary objectives of this study are to show that rapid endovascular revascularization amongst radiologically selected (small core/proximal occlusion) patients with ischemic stroke results in improved outcome compared to patients treated in clinical routine. Eligible patients will be enrolled within 12 h of last seen normal with a baseline NIHSS >5 at the time of randomization. There must be a confirmed symptomatic intracranial occlusion, based on single phase, multiphase, or dynamic CTA, at one or more of the following locations: carotid T/L, M1 MCA, or M1-MCA equivalent (2 or more M2-MCAs). All patients will receive the best standard of medical care according to modern acute stroke care guidelines (IV alteplase if <4.5 h from symptoms onset). Intervention arm consist of endovascular treatment by means of retrievable stent plus other endovascular treatment at discretion of interventionalist. This study consists of one 90-day study period for each subject. This study is in the process of enrolling participants.

Solitaire FR as primary treatment for acute ischemic stroke (SWIFT-PRIME) (ClinicalTrials.gov Identifier: NCT016557461). It is a multicentre, two-arm, prospective, randomized, open, blinded-endpoint study comparing functional outcomes (defined by mRS) in acute ischemic stroke patients who are treated with either intravenous tPA alone or intravenous in combination with Solitaire mechanical thrombectomy intervention. The sample size is up to 833 patients. Key inclusion criteria are age 18–85, prestroke functional independence, NIHSS 8–29, start of intravenous tPA within 4.5 h of onset, M1 MCA, or intracranial ICA occlusion on CTA or MRA, and target mismatch penumbral profile on multimodal CT or MR imaging. Patients allocated to the device arm will undergo mechanical thrombectomy with up to three passes of the Solitaire stent-retriever. Rapid procedure start is emphasized, within 90 min after penumbral imaging. The primary endpoint is degree of global disability at 90 days. Secondary clinical endpoints are all-cause mortality, functional independence (mRS 0–2) at 90 days, and early neurologic deficit improvement (NIHSS change at 24 h); secondary technical efficacy endpoints include revascularization/reperfusion at 24 h and infarct volume at 24 h.

THERAPY trial (A Prospective, Randomized Trial to Assess the Role of Mechanical Thrombectomy as Adjunctive Treatment to IV rtPA) (ClinicalTrials.gov Identifier: NCT01429350). THERAPY is a prospective, multicentre, randomized, concurrent controlled study. Patients from 18 to 85 years old (*n* = 582) presenting with acute ischemic stroke symptoms, an NIHSS score of at least 8 or aphasic, and eligible for intravenous rtPA with evidence of a clot at least 8 mm long in the anterior circulation from reconstructed thin-sliced non-enhanced CT are randomly assigned 1:1 to intravenous rtPA therapy alone or combined intravenous rtPA therapy and adjunctive treatment with the Penumbra System. The primary endpoints are good 90-day functional outcome and incidence of serious adverse events. Secondary endpoints include good neurological and functional outcomes at discharge and 30 days, as well as the incidence of ICH.

## Conclusion

Currently, intravenous rt-PA is the only approved treatment for acute ischemic stroke within 4.5 h from symptoms onset. However, many patients are still left undertreated, mainly due to the short time window and other contraindications for intravenous tPA. Several strategies have been developed to increase the number of treated patients. In the field of acute diagnosis, the use of multimodal neuroimaging allows physicians to evaluate not only the ischemic core but also the vessel pattern and collateral status. Concerning treatment, several molecules are being tested in randomized clinical trials with extended time windows. In this context, endovascular treatment is a promising technique that allows physicians not only to treat patients in extended time windows but also to treat patients in whom intravenous tPA has failed. Regarding endovascular treatments, a new era has emerged with new devices called stent-retrievers that have demonstrated higher rates of recanalization and clear superiority over previous devices employed in RCTs. However, the failure of recent endovascular trials to demonstrate the benefit of an endovascular approach over intravenous tPA has forced new trial designs. Several ongoing randomized clinical trials are now investigating two main research questions: the first one is whether bridging therapy is more effective than intravenous treatment alone and the second one is whether mechanical thrombectomy is more effective than the best medical treatment in patients ineligible for intravenous thrombolysis. We would therefore argue that the most advisable strategy to make progress in the field of reperfusion therapies for acute ischemic stroke is to randomize patients in well-designed clinical trials.

## Conflict of Interest Statement

The authors declare that the research was conducted in the absence of any commercial or financial relationships that could be construed as a potential conflict of interest.
